# Supplementary material for: PINK1 drives production of mtDNA-containing extracellular vesicles to promote invasiveness
Source: J Cell Biol. 2021 Oct 8;220(12):e202006049. doi: 10.1083/jcb.202006049 (PMC8641410; doi:10.1083/jcb.202006049)
Supplement: Table S1 — shows the catalog numbers of the antibodies used in the study. [file JCB_202006049_TableS1.docx]

| **Antibody recognising:** | **Western Blot Dilution** | **ELISA concentration** | **Company** | **Catalogue number** |
| --- | --- | --- | --- | --- |
| hCD63 | 1:1000 | - | BD Biosciences | 556019 |
| mCD63 | 1:1000 | - | Abcam | ab217345 |
| CD9 | 1:1000 | - | Cell Signalling Technology | 13174S |
| Flotillin | 1:1000 | - | Cell Signalling Technology | 18634 |
| β-actin | 1:5000 | - | Sigma-Aldrich | 1978 |
| Rab27A | 1:1000 | - | Abcam | 55667 |
| VDAC | 1:1000 | - | Abcam | ab14734 |
| Cyclophilin D | 1:1000 | - | Abcam | ab110324 |
| GLUD1 | 1:1000 | - | Abcam | ab153973 |
| PINK1 | 1:1000 | - | Novus | BC100-494 |
| LC3B | 1:1000 | - | Cell Signalling Technology | 2775 |
| FIP200 | 1:2000 | - | Proteintech | 17250-1-AP |
| MT1 MMP | - | 5 µg/ml | Millipore | mab3328 |
| α5 integrin | - | 5 µg/ml | BD Pharmingen | 555651 |
| active β1 integrin | - | 5 µg/ml | BD Biosciences | 09351D |
